# The Global Health System: Linking Knowledge with Action—Learning from Malaria

**DOI:** 10.1371/journal.pmed.1000179

**Published:** 2010-01-19

**Authors:** Gerald T. Keusch, Wen L. Kilama, Suerie Moon, Nicole A. Szlezák, Catherine M. Michaud

**Affiliations:** 1 Department of International Health, Boston University School of Public Health, Boston, Massachusetts, United States of America; 2 African Malaria Network Trust, Dar es Salaam, Tanzania; 3 Sustainability Science Program, John F. Kennedy School of Government, Harvard University, Cambridge, Massachusetts, United States of America; 4 Harvard Initiative for Global Health, Harvard University, Cambridge, Massachusetts, United States of America; London School of Hygiene and Tropical Medicine, United Kingdom

## Abstract

In the third in a series of articles on the changing nature of global health institutions, Gerald Keusch and colleagues examine institutional arrangements for malaria research.

*This is the third in a series of four articles that highlight the changing nature of global health institutions.*

## Introduction

Conducting basic research, translating it into the development of new health tools, and delivering products to patients in need of them are core functions of an effective global health system [1]. Yet performing these functions is a particular challenge for diseases that primarily affect the poor in low-income countries, partly because efforts to understand diseases and develop tools to combat them are often detached from efforts to deliver interventions. For malaria, the global health system has evolved over the past century to integrate better the research, development, and delivery (R&D&D) of new products to treat and control the disease. This article traces that evolution and extracts lessons applicable to the many new challenges currently facing the global health system.

Historically, global investment in malaria research has been disproportionately small relative to its disease burden. Research funding in endemic countries was seriously limited by resource and capacity constraints, while funding agencies in industrialized countries were primarily concerned with domestic health issues, with the important exception of military needs to control malaria. Recently, however, global malaria R&D investments have increased dramatically, from an estimated $84 million in 1993 [2] to $323 million in 2004 [3], with a new focus on malaria's impacts on people in endemic countries.

In malaria control, there has been a concomitant shift from time-limited, centralized efforts—often relying on single interventions—toward a more decentralized, continuous effort using multiple approaches. Malaria is no longer seen primarily as a biomedical problem, but rather as a complex ecological system in which humans, mosquitoes, and parasites are interconnected. Malaria has also increasingly been characterized as a “global” and regional rather than a national or local problem. This has led to changed concepts of governance. Such governance has changed in two ways: (1) from an essentially “top-down” process from international to national or local players to an active interplay between local and global players, and (2) from a system that centered on the World Health Organization (WHO), with little attention to national governments in endemic countries, to one in which state and non-state actors cooperate across multiple dimensions, emphasizing inclusion and engagement of local communities. Today, for the first time, the principal constraints to malaria control may be more political and managerial than financial or technical.

This article explores the changing global health system for malaria research and the delivery of research products to those at risk, including the organizations and actors involved, and the arrangements that govern their interactions (for more about these actors and arrangements, see the first article in this four-part series [1]). Following Alilio and colleagues [4], we have divided the evolution of malaria R&D&D into three periods (Table 1); although these divisions are somewhat arbitrary, they highlight major shifts in the system's development. Finally we address the lessons learned and speculate about the future.

**Table 1:** Evolution of institutional arrangements for malaria R&D.

Phase	Purpose of R&D Institutions	Targeted End-Users	Funding	Targeted Diseases
**I: Late Nineteenth Century through the 1950s**	National public goods	Industrialized countries	Public, private	Malaria, yellow fever
**II: 1960s–80s**	International health programs (e.g., TDR, Fogarty International Center, Rockefeller Foundation)	Developing countries	Public, philanthropic	Malaria and other tropical infectious diseases
**III: 1990s–2000s**	Global health partnerships neglected disease R&D (e.g., PDPs)	Developing countries	Public, philanthropic, private sector	Malaria, tuberculosis, HIV/AIDS and neglected tropical infectious diseases
**IV: The Future**	Global public goods for global health	Global	Public, philanthropic, private sector	All types

## Phase I. Late Nineteenth Century through the 1950s: National Public Goods

### R&D

The early driver of malaria research was the desire of the European colonial powers to protect their own nationals and the economic interests in their colonies. This investment led to many discoveries, including identification of the cause, vector, and transmission cycle of malaria. Later, when malaria debilitated allied soldiers in World War II (WWII), military needs drove malaria R&D. None of the principal malaria medicines of the twentieth century would have been discovered without military R&D [5]–[8]. Even insecticide-treated bed nets (ITNs) [9] and household spraying with DDT were used effectively by the allied militaries in WWII [6],[10].

During this long period, innovation followed a distinct trickle-down pattern. Researchers in the North produced knowledge to serve their own national needs, and only later was it applied for the benefit of low-income countries. While these R&D efforts ultimately created global benefits, the institutions that guided and benefited from the research were in rich countries. The drawback for low-income countries was that tools developed for militaries of the North were not necessarily well-suited for civilians in the South. Cost was not a major issue for the North, and because antimalarial drugs were targeted at adults, testing in children was a low priority, although children account for most malaria deaths. As the US Military Infectious Diseases Research Program recently pointed out, “Preventing death in children and keeping soldiers healthy and effective are distinct goals requiring different research strategies” [11].

### Delivery

The association between swamps, mosquitoes, and malaria has long been appreciated [12]. By the time of the Roman Empire, bed nets, decoy animals to attract mosquitoes, swamp drainage, and housing prohibitions in mosquito breeding areas were used to control malaria. The clustering of “marsh fever” among those living near smelly swamps led to the miasma theory of disease, that foul “mala aria” (bad air in Italian, from which the name malaria derives) from decomposed matter (miasmas) was the cause. Efforts at control were local and often misguided, yet sometimes effective, for example drainage of swamps and closing of mill ponds in the US in the nineteenth century.

Evidence-based systematic attempts to control malaria at a population scale date from the beginning of the twentieth century, and were based on the understanding of the transmission cycle and recognizing quinine's therapeutic value. Control programs were used in large, expensive works projects threatened by malaria (and yellow fever) and targeted at workers and managers from industrialized countries, such as the Suez and Panama Canal projects. Multiple strategies were adopted, including manual clearance of mosquito larvae, removal of breeding sites, leveling and oiling of roads to eliminate water pools, use of clothing to prevent mosquito bites at dusk, burning of pyrethrum indoors, larviciding with chemicals, treatment with quinine, use of window screens, and collection of indoor resting mosquitoes post-feeding. These strategies were effective in the limited scope of the effort. In the late 1930s, Fred Soper of the Rockefeller Foundation and 40,000 workers in Brazil successfully eradicated *Anopheles gambiae*, which had recently been imported, using pyrethrum spraying, larviciding with Paris Green (copper acetoarsenite), and elimination of breeding sites [13].

In 1946, the US Centers for Disease Control (CDC) was established in Atlanta, Georgia as the successor to the WWII Malaria Control in War Areas Agency, primarily to eradicate malaria in the southern states. According to a history of the CDC, “Pursuit of malaria was by far the most absorbing interest of CDC during its early years, with over 50 percent of its personnel engaged in it” [14]. Malaria transmission in the US was eliminated.

## Phase II. 1960s–1980s: The International Health Perspective

Phase I had involved nationally focused programs concerned with domestic social well-being (malaria in the southern US), economic gain (the canal projects), or military needs (wars in malaria zones). But subsequent years witnessed a phase of internationalization in public health, with rapid decolonization, the launch of national foreign aid initiatives amidst heightened Cold War tensions, and new faith in the potential of science and technology.

### R&D

In phase II, the relevant actors were increasingly viewing the world as interdependent [15], with greater emphasis on international health needs. The Special Programme for Research and Training in Tropical Diseases (TDR) was established within WHO in 1975, and played a key role in building malaria research capacity in developing countries, particularly in Africa where few malaria researchers existed at the time of political independence for the former colonies. TDR also established international networks of academic centers for tropical disease research, a model that the public–private product development partnerships would later emulate [16]. These national–international partnerships proved to be essential for the development of critical new tools, including artemisinin combination therapy and ITNs still in use today [9],[17],[18]. During this era the private foundations reemerged as a force, for example the Great Neglected Diseases (GND) of Mankind Biomedical Research Network launched by Kenneth Warren of the Rockefeller Foundation [19]. Catalyzed by GND funds, a stream of young scientists from developing and developed nations were attracted to work with established researchers in this global network on problems such as malaria in the laboratory and the field.

Compared to the previous period, institutions for R&D were broader in scope, more international, and targeted low-income country needs. However, as the Rockefeller Foundation's Tim Evans later noted, GND produced “improved basic knowledge about poorly understood tropical diseases….[but] no explicit strategy to translate new knowledge into drug or vaccine development” [20]. By the late 1980s the GND was winding down, TDR was seriously underfunded for its broad mandate, and the pharmaceutical industry had largely withdrawn from tropical infectious disease research. The existing R&D system could not meet the vast health needs of low-income countries. The research enterprise had simultaneously succeeded and failed.

### Delivery

In 1955, based on the wartime success of DDT, WHO initiated an ambitious attempt to eradicate malaria by eliminating the vector. However, by 1969 the Global Malaria Eradication Programme (in fact it was never global, excluding much of sub-Saharan Africa from the outset) was considered to have failed. The program had nevertheless achieved considerable success in 25 countries in Africa, Asia, Europe, North America, and the Caribbean, primarily relatively rich and island countries and a few poor countries with good health infrastructure and seasonal malaria [21]. There were many reasons to give up the effort, including donor funding fatigue, local resistance to the imposition of control measures, insecticide resistance, and the difficulty of mosquito eradication in many ecosystems. Efforts reverted to control [22], and with the momentum for primary health care and the 1978 Alma Ata declaration calling for “Health for All” by the year 2000, malaria control was incorporated into primary care programs.

With the loss of visibility, combined with waning global interest and dwindling funding for research and control, malaria was soon overshadowed by the emerging HIV/AIDS epidemic in the 1980s. Between 1975 and 1994, malaria control was financed mostly as bilateral assistance to endemic countries, with yearly contributions of less than $20 million, for an estimated $364 million over 20 years. The impact of the “Silent Spring” movement and the near total cessation of DDT production [23], accompanied by rapid spread of resistance to the nearly ideal antimalarial chloroquine, contributed to the resurgence of malaria, including in places where it had formerly been controlled. Malaria research and control had itself become a neglected initiative.

## Phase III. 1990s to the Present: Global Health and Malaria Research and Control

Many factors have led to the consideration of health as a global imperative over the past two decades, particularly the disparate burden of HIV in poor nations and the AIDS activist movement, which revived a human rights approach to health care. With these changes in the value system and increasing attention to the concept of global public goods, malaria R&D and delivery have become priorities again.

### R&D

In 1990, the independent Commission on Health Research for Development argued in its seminal report, *Health Research-Essential Link to Equity in Development*, that research had long been “under-recognized and neglected” as a tool to mitigate growing global inequities in public health [24]. With increasing globalization of trade, travel, information, and disease, health in general and R&D in particular were increasingly framed as “global” rather than “international,” concerned with “the health needs of the people of the whole planet above the concerns of particular nations” [25]. This change also underscored “the growing importance of actors beyond governmental or intergovernmental organizations” [24]. The report of the WHO Ad Hoc Committee on Health Research Relating to Future Intervention Options set priorities for global health research and recommended an approach to allocate research funding [26]. Because confidence in the leadership at WHO among key global players was at an all-time low, an independent organization, the Global Forum for Health Research, was established in Geneva to catalyze and monitor investments in research relevant to the world's poorest people [27].

Malaria was a good example of a neglected disease in 1990, as both public and private actors had largely retreated from malaria research, even though drug-resistant malaria was spreading across the globe. In 1996, Harold Varmus, then Director of the US National Institutes of Health (NIH), concluded that malaria R&D merited increased funding because of its global impact and the potential for scientific progress with increased funding. The same year, the UK-based Wellcome Trust reported on the domination of malaria research by scientists from the North [2]. In part because WHO was not deemed to have sufficient scientific depth or resources to address these disparities, the Multilateral Initiative on Malaria (MIM) was established in 1998 as a joint effort of northern country health research and bilateral aid agencies [28]. MIM rapidly improved channels for information flow between researchers in the North and South through data sharing and internet-based library access; established a repository for patient-, parasite-, and vector-derived chemical entities and genomes for research; provided research funds for African scientists through TDR; and initiated a regular Pan African Malaria conference to bridge the malaria research and control communities. The MIM Secretariat, based successively at the Wellcome Trust, the Fogarty International Center at NIH, and the Karolinska Institute, moved to its first African home in Tanzania in 2006, although securing long-term financial support remains a vexing problem. Enlightened leadership and commitment from the elite science funding agencies was the essential catalyst behind these changes.

By the late 1990s, the increasing self-confidence of senior African scientific leaders and maturation of young African malaria researchers into senior leaders, and recognition of their contributions to knowledge generation, placed them at the center of research planning and progress in malaria. Trainees in basic sciences, entomology, epidemiology, biostatistics and bioinformatics, sociology, behavioral sciences, and public health now could play key roles and become deeply involved in institutional leadership and management. The creation of MIM also pushed forward the visibility of malaria as a global problem, engaged leading research funding institutions in the North, and supported a global research network to link research to control. These actions laid the immediate groundwork for the launch of public-private–product development partnerships (PDPs) for malaria.

With the support of major foundations, PDPs emerged in the 1990s to address a glaring failure of existing institutional arrangements for R&D—that market-incentives had proven insufficient to drive investment in new tools for neglected diseases [29]. PDPs have redefined roles and expectations, with the public sector playing a stewardship and funding role, the private sector contributing materials and know-how, and private philanthropy investing a significant share of the funds. New PDPs doing malaria research have been created, including Medicines for Malaria Venture (MMV), Malaria Vaccine Initiative (MVI), Drugs for Neglected Diseases Initiative (DNDi), and the Institute for OneWorld Health (iOWH). Thus far, two new fixed-dose combination malaria treatments based on artemisinin derivatives (DNDi) [30], a lower-cost synthetic method to produce artemisinin (iOWH) [31], and a licensed pediatric formulation of an artemisinin combination drug and a pipeline of new compounds in development to address emerging drug resistance (MMV) [32] have resulted. Though relatively new, PDPs have reinvigorated product development for malaria and other neglected diseases [33]. Furthermore, by placing affordability and accessibility at the center of their missions, they promote the concept of health R&D as a global public good [34]. Importantly, PDPs are explicitly expected to develop products well-adapted for use in low-income countries [35]. Nevertheless, the PDPs are relatively young, and it remains to be seen if they can efficiently deliver on their early promise over the long haul.

Local initiatives are now apparent as well. The African Malaria Network Trust [36] and the Malaria Clinical Trials Alliance [37] are African-led initiatives to strengthen malaria-related R&D capacities in Africa. They collaborate with northern partners and malaria PDPs to support African research institutions to develop products up through Phase III clinical trials. This reflects the recognition of African malaria scientists with the skills to conduct basic and clinical research and compete for funding, and reinforces the new norm that neglected disease research should involve endemic-country scientists and be targeted to meet low-income country needs.

### Delivery

In 1992, the WHO Ministerial Conference on Malaria in Amsterdam [38] outlined a broad set of measures to reduce the burden of malaria, including early diagnosis and treatment, selective and sustainable preventive measures, early identification of epidemics and rapid responses to contain them, and strengthening of local capacities in basic and applied research. Much of this agenda was supported by northern research agencies, not WHO. To reestablish a central role for WHO, the newly elected Director General, Gro Bruntland, in 1998 established the Roll Back Malaria (RBM) Partnership as a “Cabinet Project” reporting directly to her [39]. It signaled a new order of business at WHO—a global program partnership—responding to the widely held belief that malaria could not be controlled by governments and WHO alone but needed multiple public and private partners to succeed. The World Bank, UNICEF, DFID, USAID, foundations, NGOs, and the private sector quickly joined RBM, together with national governments and their malaria control agencies.

RBM's mandate was to seek greater funding, raise awareness of malaria as a global problem, harmonize activities of the partners and support development of effective national programs. However, heavy-handed management by the Secretariat at WHO led to dissatisfaction with progress among the partners and with the manner in which they were being engaged. An external evaluation, required by the partners, damned with faint praise the accomplishments of the first four years, noting that advocacy was not supported by data, decision-making was inefficient, accountability within the Partnership was lacking, reductions in the malaria burden had been “slower than anticipated,” countries “receive inadequate and sometimes inconsistent technical advice,” and insufficient attention was given to “multi-sectoral approaches, particularly as regards private sector activity” [40]. Since then, RBM's performance has improved. RBM's recently issued Global Malaria Action Plan outlining strategies, costs, goals, and timelines is a major accomplishment, with multiple partner inputs. RBM is commissioning an independent evaluation to appraise the “governance; management; ability to convene, coordinate and harmonize RBM partners and stakeholders; and its impact on malaria efforts at country level” [41].

The Global Fund to Fight AIDS, Tuberculosis and Malaria (GFATM) was founded in 2002 as a new international financing mechanism for these three diseases and to harness the capacities of public, private, and civil society actors at both global and national levels [42]. GFATM was based on the premise that success depended on the involvement of multiple state and non-state actors. It was explicitly created as a public–private entity outside of and independent of the UN system. Furthermore, the concentration of funds from multiple public and private sources in GFATM (which totaled $1.6 billion for malaria control between 2002 and 2007) was intended to decrease prior fragmentation of funding schemes. Despite increasing multilateralism from many global health actors, the US has been a reluctant partner in GFATM since its founding, preferring to invest most of its significantly increased commitment through bilateral programs, such as the President's Emergency Fund for AIDS Relief (PEPFAR) and the President's Malaria Initiative (PMI). Total US pledges to GFATM from its inception through January 2009 amount to $4 billion. The Obama administration's budget request for 2010 includes a 36% increase in malaria support, a 2.5% increase for PEPFAR, but no change in funding for GFATM, thus continuing the major emphasis on bilateral program support.

## Phase IV. The Future: Lessons Learned and Global Public Goods for Global Health

The past 30 years has witnessed significant shifts in the types of actors and the roles they play in malaria research and control, with gradually increasing integration of the R&D&D communities. With these changes, a number of new modes of operation have been established that seem certain to continue, such as: (1) a more central role for endemic-country researchers in an increasingly globalized research system; (2) direct funding to local researchers and institutions; (3) the involvement of affected communities not only as targets of interventions but as co-producers of results; (4) new actors taking on tasks formerly vested in WHO; and (5) new PDPs to drive research to unmet needs and new product development. These developments bode well for achieving the prospects for new, effective, adapted, and affordable tools for malaria

A new challenge to the global health system is the recent decision by the Bill and Melinda Gates Foundation, in addition to its support of malaria PDPs, to place malaria eradication back on center stage [43]. Not all experts agree that malaria eradication is feasible or desirable [44]. Regardless of the validity of the criticism, it is necessary to continue to develop and apply new tools to eliminate malaria as a significant public health problem, as disease reduction is the necessary antecedent to any attempt at eradication of the parasite.

New mechanisms for partnerships among global and national organizations have pioneered new approaches to financing and governance of programs, such as GFATM and RBM, along with major bilateral investments, such as PEPFAR and PMI. In addition, the interests of the science community now connect to product development to tackle growing drug and insecticide resistance. These innovations have focused on neglected infectious diseases that by definition affect *only* developing countries. Such innovations leave the question unanswered of who contributes to and who benefits from R&D for diseases that affect *all* countries, such as noncommunicable diseases, including cancer, cardiovascular disease and stroke, diabetes, and obesity [45]–[50] The challenge of building effective new R&D&D arrangements in the twenty-first century for all health needs of all people should be informed by past developments in malaria. The most relevant developments to draw upon are the challenges of filling the institutional gaps within the global health system to link R&D with delivery; of effectively connecting local and international researchers; and finally of ensuring support for the generation of global public goods for global health. A step in that direction has recently been taken by the leading national research agencies of a number of developed and developing nations. These have come together to form the Global Alliance for Chronic Diseases [50], with a pledge to invest in research in a coordinated manner, and to scale up promising interventions to achieve targeted goals of disease reduction.

## Conclusions

In this paper, we have reviewed the century-long effort to research malaria, to develop tools for control, and to implement them. It is clear from our review that support for and inclusion of local research institutions in global health research is essential to develop well-adapted health tools and to strengthen collaborations between global and local actors in implementation. Such support and inclusion is a necessary precursor to the emergence of stronger and more integrated global research, development, and delivery, which we have termed the R&D&D system. The role of WHO in this global system must evolve as a partnership with other actors.

Building an effective global health system takes times. It required decades to build up research capacity in malaria-endemic countries to the present level, when local researchers can play an integral role in malaria R&D&D. Investments in capacity building in other relatively neglected areas, such as noncommunicable diseases, must begin today if we expect similar dividends in the future.

R&D must connect closely to the challenges of implementation. The historical divide between academic research, industry development, and those who implement in the real world cannot continue if “acting in time,” translating knowledge into action, is a critical goal. Those in the R&D world must understand what the control community has to deal with, and the latter need to know what is in the R&D pipeline in order to identify the delivery constraints that must be solved.

Enlightened leadership within organizations comes with a commitment to scaling up the level of R&D and capacity-building investments, harnessing the potential gained from connecting researchers in the North and the South, and articulating the messages to decision makers and the general public to gain support. The new Global Alliance for Chronic Disease appears to have heard the message, as these issues are highlighted in its mandate.

Finally, the case study of malaria suggests that a multiplicity of partnership models is useful, particularly for diseases that require multiple interventions and continuing R&D. The global health system of the future must identify ways to include those who suffer from diseases, those who contribute to R&D, and those who deliver interventions, sharing the responsibility to link better knowledge with action for those in need.

## Abbreviations

CDC: US Centers for Disease Control
DDT: dichloro-diphenyl-trichloroethane
DFID: Department for International Development (UK)
DNDi: Drugs for Neglected Diseases Initiative
GFATM: Global Fund for AIDS, Tuberculosis and Malaria
GND: Great Neglected Diseases Biomedical Research Network
IoWH: Institute for One World Health, ITN, insecticide-treated bed net
MIM: Multilateral Initiative on Malaria
MMV: Medicines for Malaria Venture
MVI: Malaria Vaccine Initiative
NGO: non-governmental organization
NIH: US National Institutes of Health
PDP: Product Development Partnership
PEPFAR: President's Emergency Program for AIDS Relief
PMI: President's Malaria Initiative
R&D: research and development
R&D&D: research and development and delivery
RBM: Roll Back Malaria
UNICEF: United Nations Children's Fund
USAID: United States Agency for International Development
WHO: World Health Organization
